# Impact of Ganglionated Plexus Ablation on Early Recurrence After Thoracoscopic Atrial Fibrillation Ablation: A Post Hoc Analysis of the AFACT Study

**DOI:** 10.1111/jce.70517

**Published:** 2026-10-06

**Authors:** Zhenyu Dong, Marc M. Terpstra, Jolien Neefs, Nicoline W.E. van den Berg, Femke R. Piersma, Sebastien P.J. Krul, Wouter R. Berger, Wim‐Jan P. van Boven, Jonas S.S.G. de Jong, Ruben Coronel, Joris R. de Groot, Antoine H.G. Driessen

**Affiliations:** ^1^ Departments of Clinical and Experimental Cardiology, Heart Center Amsterdam UMC, location University of Amsterdam Amsterdam the Netherlands; ^2^ Amsterdam Cardiovascular Sciences, Heart Failure and Arrhythmias Amsterdam the Netherlands; ^3^ Department of Cardiology Heart Center, Isala Zwolle the Netherlands; ^4^ Department of Cardiology Onze Lieve Vrouwe Gasthuis (OLVG) Amsterdam the Netherlands; ^5^ Department of Cardiothoracic Surgery Heart Center, Amsterdam UMC, location University of Amsterdam Amsterdam the Netherlands

**Keywords:** atrial fibrillation, early recurrence, GP ablation, late recurrence, substrate mechanisms

## Abstract

**Background:**

Early recurrences of atrial fibrillation (AF) after ablation are often considered transient. However, they may be caused by a combination of temporary and permanent proarrhythmogenic factors.

**Objective:**

We evaluated whether adjunctive GP ablation was associated with the timing and clinical implications of early AF recurrence, and explored the hypothesis that autonomic mechanisms contribute to early recurrence.

**Methods:**

Using data from the randomized AFACT study, we compared patients with advanced AF who underwent stand‐alone thoracoscopic ablation with or without GP ablation. Early recurrence was defined as AF or atrial tachycardia recurrence within 90 days postoperative. Cox models were used to assess time‐dependent effects and identify clinical predictors.

**Results:**

A total of 235 patients were included in the current analysis. Seventy‐one patients had early recurrence: 36 in the GP ablation group and 35 in the non‐GP ablation group (*p* = 0.730). Of these, 45 (63%) occurred within the first 30 days. Patients in the GP ablation group had their first recurrence later than those in the non‐GP group (24 [14–34] vs. 14 [11–24] days, *p* = 0.046). In the GP ablation group, markers of arrhythmogenic substrate (i.e., age, left atrial volume index, diabetes, CHA_2_DS_2_‐VASc score, and persistent AF) were associated with early recurrence. Additionally, early recurrence was associated with late recurrence in the GP ablation group, but not in the non‐GP ablation group.

**Conclusion:**

Adjunctive GP ablation was associated with later onset of first early recurrence, while the overall early recurrence rate was not reduced. In the GP ablation group, early recurrence was associated with subsequent late recurrence. These exploratory findings are compatible with a model in which early recurrence reflects both transient autonomic triggers and pre‐existing substrate, but this mechanism remains hypothesis‐generating.

**Clinical Trial Registration:** ClinicalTrials.gov Identifier: NCT01091389.

## Graphical abstract

Adjunctive ganglionated plexus (GP) ablation did not reduce early AF recurrence after thoracoscopic AF ablation but was associated with a later first recurrence. In the GP‐ablation group, early recurrence was associated with late recurrence; this exploratory finding is compatible with, but does not establish, a greater contribution of pre‐existing substrate.

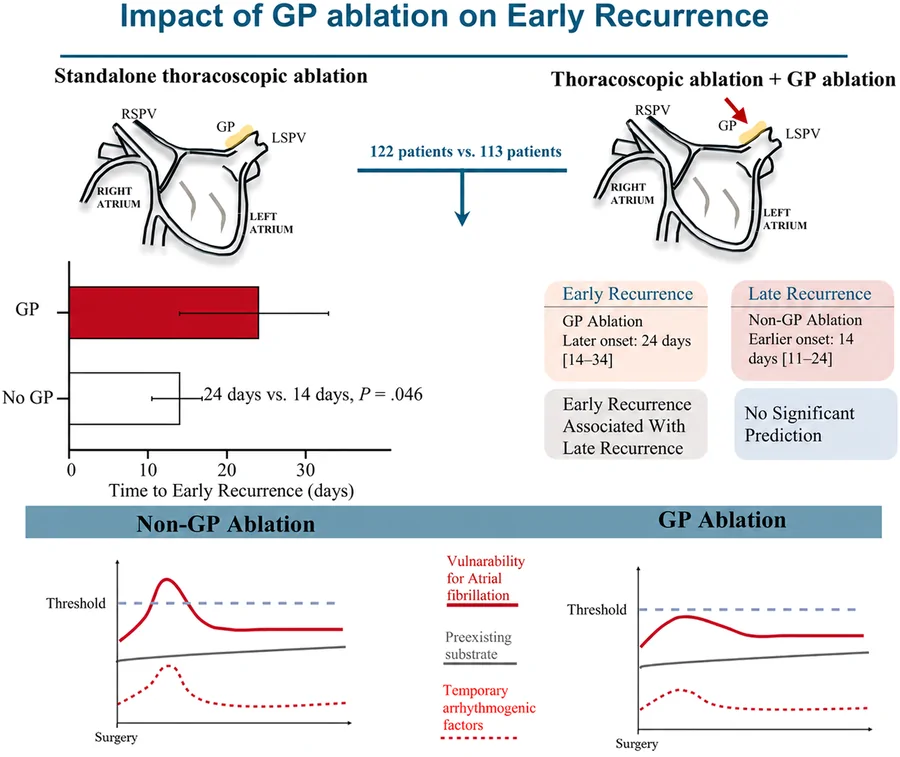

**Abbreviations**
AF: atrial fibrillation
AFACT: atrial fibrillation ablation and autonomic modulation via thoracoscopic surgery
CI: confidence interval
GP: ganglionated plexus
GSEA: gene set enrichment analysis
HR: hazard ratio
LAVI: left atrial volume index
PVI: pulmonary vein isolation

## 1 Introduction

Atrial fibrillation (AF) substantially impairs quality of life and imposes a significant burden on patients and healthcare systems [1]. Pulmonary vein isolation (PVI) is considered an effective treatment to reduce symptoms and improve patients' quality of life [1]. Early recurrence of AF within the first 90 days post‐procedure (blanking period) following catheter or thoracoscopic PVI is common, occurring in up to 61% of patients, but does not necessarily predict long‐term treatment failure [2, 3]. Consequently, early AF recurrences during the blanking period are not considered procedural failures and are often attributed to transient, non‐substrate‐related factors such as acute inflammation and autonomic imbalance [2, 4]. However, early recurrence has also been shown to increase the risk for late AF recurrence [2, 5]. Therefore, early AF recurrences after ablation may reflect temporary triggers induced by the procedure, an underlying arrhythmogenic substrate, or a combination of both.

Ganglionated plexus (GP) ablation and other forms of autonomic modulation have been investigated as approaches to modify vagal and sympathetic inputs to the atria [4, 6, 7, 8]. In the Atrial Fibrillation Ablation and Autonomic Modulation via Thoracoscopic Surgery (AFACT) study, patients with advanced AF were randomized to GP ablation or no GP ablation in addition to thoracoscopic PVI [9]. Although the main trial showed no long‐term efficacy and increased morbidity with GP ablation, arguing against its use in routine practice, the randomized design enabled us to explore whether GP ablation was associated with different timing and prognostic implications of early AF recurrence, with reduced confounding compared with observational comparisons [9, 10]. This post hoc analysis of the AFACT study aimed to assess the association between GP ablation and early AF recurrence following thoracoscopic PVI, and to generate hypotheses regarding mechanisms underlying early recurrence.

We therefore explored a stage‐dependent hypothesis of AF recurrence, whereby early recurrence may reflect both autonomic triggers and pre‐existing atrial substrate remodeling, whereas later recurrence may be more strongly related to progressive substrate remodeling. Accordingly, early recurrence persisting after adjunctive GP ablation might be more indicative of a pre‐existing arrhythmogenic substrate.

## 2 Methods

### 2.1 Study Design

We performed a post hoc exploratory sub‐analysis of the single‐center randomized AFACT study (NCT01091389), which compared stand‐alone thoracoscopic ablation with or without adjunctive GP ablation in patients with advanced AF, with 1‐year follow‐up [9]. Adults aged 18‐80 years undergoing thoracoscopic AF ablation for advanced AF, having failed ≥ 1 Class Ic or III antiarrhythmic drug and with an expected survival ≥ 2 years, were eligible. Advanced AF was defined as persistent AF, or enlarged left atria (left atrial volume index (LAVI) ≥ 33 mL/m^2^ [2], or prior failed catheter ablation [9]. Written informed consent was obtained from all patients. The inclusion and exclusion criteria have been described previously [9]. This report adheres to the STROBE guidelines.

### 2.2 Surgical Procedure

The surgical procedure and GP ablation protocol have been described in detail previously [11]. In brief, bilateral video‐assisted thoracoscopy was performed, and all patients underwent PVI. For persistent AF, a roof line was added between the pulmonary veins, along with a trigone line to the left fibrous trigone. All linear lesions were tested for bidirectional block as previously described [12]. Patients randomized to GP ablation first underwent right‐sided GP ablation guided by high‐frequency stimulation (16 Hz, 1.0 ms pulse width, 1–25 mA), with a positive response defined as ≥ 50% R‐R interval prolongation. If no response occurred, anatomically guided ablation was applied. Subsequently, superior and inferior left‐sided GPs were ablated, the ligament of Marshall was dissected, and high‐frequency stimulation was repeated for all four major GPs to confirm elimination of vagal responses, with additional ablation performed as required. During surgery, the left atrial appendage was excised in all patients [9, 13], and a subset of specimens was randomly selected for bulk RNA sequencing and subsequent gene set enrichment analysis (GSEA), as detailed in the Methods section of the Supporting Information S1: Supplementary Material.

### 2.3 Postoperative Rhythm Monitoring and Endpoint Definition

Follow‐up visits were scheduled at 10 days post‐discharge and 3, 6, 9, and 12 months, with standard electrocardiography and 24‐h Holter monitoring according to the study protocol. Symptomatic patients were encouraged to obtain additional rhythm recordings as needed [9]. AF recurrence was defined as any atrial tachyarrhythmia lasting ≥ 30 s, documented by electrocardiography, Holter monitoring, or device electrograms (pacemaker or implantable cardioverter‐defibrillator). Early recurrence was defined as AF or atrial tachycardia occurring within 90 days following the ablation, while late recurrence was defined as recurrence beyond this period [14]. All patients received colchicine 0.5 mg once daily for 30 days from the first postoperative day to prevent pericarditis. Antiarrhythmic drugs were continued during the blanking period and discontinued after 3 months.

### 2.4 Statistical Analysis

Categorical variables were compared using *χ* ^2^ or Fisher's exact tests, and continuous variables using independent‐samples *t*‐tests or Mann–Whitney *U* tests, as appropriate. Data are reported as mean ± standard deviation or median (interquartile range). Time‐to‐event outcomes were analyzed using Kaplan–Meier curves and Cox proportional hazards models, with hazard ratios (HRs) and 95% confidence intervals (CIs) reported. Loss to follow‐up was treated as right‐censoring. Due to the small number of such events, competing risk analysis was not performed. Missing covariate data were handled using multiple imputation by chained equations. Time‐varying treatment effects were assessed using a Cox model with a time‐by‐treatment interaction. Multivariable Cox proportional hazards models were fitted, including age, LAVI, diabetes mellitus, AF type, and GP ablation, with prespecified interaction terms for age × GP and LAVI × GP. Covariates were selected a priori based on clinical relevance to AF, and interaction terms were retained to test whether GP ablation modified their associations with early recurrence. Given the exploratory post hoc design, no formal correction for multiple comparisons was applied; therefore, *p* values from subgroup and interaction analyses should be interpreted as exploratory. All analyses were performed in R version 4.3.3; *p* < 0.05 was considered statistically significant. Central Illustration 1.

## 3 Results

### 3.1 Baseline Characteristics

Among the 240 patients enrolled in the AFACT study, 117 were randomized to the GP ablation group, and 123 to the non‐GP ablation group. The procedure was aborted in two patients from the GP group. Three patients (two from the GP group, one from the non‐GP group) were excluded due to uncertain early recurrence timing. Left ventricular ejection fraction was missing in 6 patients, and indexed left atrial volume (LAVI) was missing in 1 patient. During the 90‐day follow‐up period, one 76‐year‐old male in the GP group died on postoperative day 22 due to sepsis and multiorgan failure. A total of 235 patients were included (mean age 59.4 ± 8.2 years; 170 [72.3%] male) (Table 1). Baseline characteristics were generally similar between patients with and without early recurrence (Table 1). Patients with early recurrence had a longer duration of AF than those without early recurrence (6 [3–9] vs. 4 [2–7] years, *p* = 0.044).

**Table 1:** Baseline characteristics of the study population.

	Overall (*n* = 235)	No early recurrence (*n* = 164)	Early recurrence (*n* = 71)	*p*
Age, years	59 (8)	59 (8)	61 (7)	0.109
Sex, female	65 (27.7)	47 (28.7)	18 (25.4)	0.718
Body mass index (kg/m^2^)	27.33 (3.87)	27.34 (3.98)	27.30 (3.63)	0.942
Left atrial volume index (ml/m^2^)	41.8 (12.6)	41.4 (13.4)	42.8 (10.5)	0.448
Left ventricular ejection fraction (%)	50.0 (10.4)	50.6 (10.0)	48.6 (11.1)	0.173
Previous catheter ablation	51 (21.7%)	34 (20.7%)	17 (23.9%)	0.707
History of myocardial infarction	11 (4.7)	9 (5.5)	2 (2.8)	0.512
Duration of atrial fibrillation (years)	4 (2–8)	4 (2–7)	6 (3‐9)	0.044
Paroxysmal atrial fibrillation	96 (40.9)	74 (45.1)	22 (31.0)	0.060
CHA_2_DS_2_‐VASc score = 0	67 (28.5)	48 (29.3)	19 (26.8)	0.815
History of congestive heart failure	12 (5.1)	8 (4.9)	4 (5.6)	0.757
History of hypertension	101 (43.0)	70 (42.7)	31 (43.7)	1.000
History of diabetes mellitus	15 (6.4)	9 (5.5)	6 (8.5)	0.395
History of stroke, TIA, or embolism	20 (8.5)	14 (8.5)	6 (8.5)	1.000
History of vascular disease	24 (10.2)	17 (10.4)	7 (9.9)	1.000
Age ≥ 65 years	65 (27.7)	41 (25.0)	24 (33.8)	0.220
Age ≥ 75 years	2 (0.9)	2 (1.2)	0 (0.0)	1.000
Antiarrhythmic drugs				
Class IA	6 (2.6)	3 (1.8)	3 (4.2)	0.370
Class IC	78 (33.2)	56 (34.1)	22 (31.0)	0.748
Class II	120 (51.1)	79 (48.2)	41 (57.7)	0.228
Class III	94 (40.0)	69 (42.1)	25 (35.2)	0.400
Class IV	31 (13.2)	25 (15.2)	6 (8.5)	0.229

### 3.2 Time‑Dependent Effect of GP Ablation on Early Recurrence

Early recurrence occurred in 36/113 (31.9%) in the GP group and 35/122 (28.7%) in the non‐GP group. Among all early recurrences, 45/71 (63%) occurred within 30 days. Among patients who experienced early AF recurrence (*n* = 71), the first recurrence occurred later in the GP ablation group than in the non‐GP group (24 [14–34] vs. 14 [11–24] days; *p* = 0.046; Figure 1, 2). A time‐dependent Cox model with a treatment × log(time) interaction suggested a possible time‐varying treatment effect (*χ* ^2^ = 3.70, df = 1, *p* = 0.054). The estimated HR was < 1 during the first ~2 weeks, crossed unity around day 14, and increased thereafter, a pattern compatible with a possible transient early effect (Supporting Information S1: Figure S1). GP ablation was not associated with a lower cumulative incidence of early recurrence (0–90 days; HR 1.09; 95% CI 0.68–1.73; *p* = 0.730; Figure 1A).

**Figure 1:**
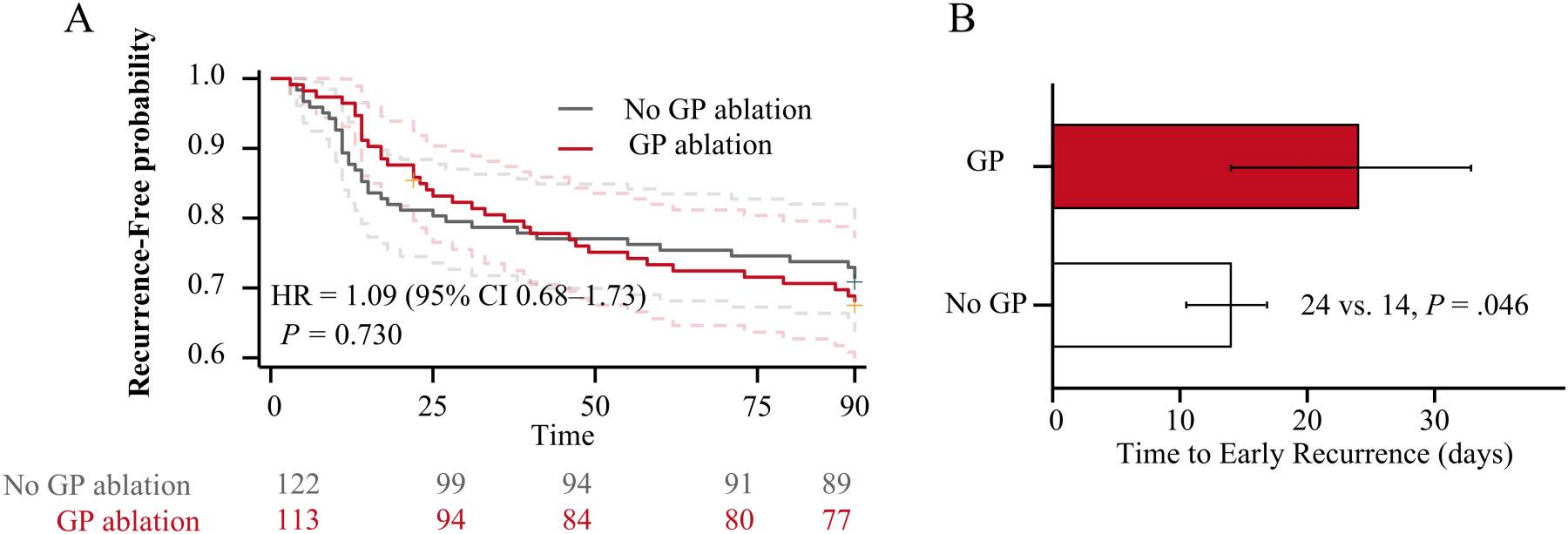
Early recurrence after thoracoscopic ablation with or without GP ablation. (A) Kaplan–Meier curves of recurrence‐free probability during the 0–90‐day blanking period comparing No GP ablation vs GP ablation. (B) Time to first early recurrence among patients with an event, by treatment group. Bars represent median (interquartile range).

### 3.3 Clinical Predictors of Early Recurrence and Effect Modification by GP Ablation

In univariate Cox regression analysis, several clinical factors were associated with early recurrence among patients who underwent GP ablation (*n* = 113, Figure 2), including age (HR 1.07, 95% CI 1.02–1.12, *p* = 0.004), persistent AF (HR 2.69, 95% CI 1.18–6.15, *p* = 0.019), LAVI (HR 1.03, 95% CI 1.01–1.06, *p* = 0.011), diabetes mellitus (HR 2.98, 95% CI 1.05–8.45, *p* = 0.040), and CHA_2_DS_2_‐VASc score (HR 1.30, 95% CI 1.02–1.65, *p* = 0.032). Comparable associations were not observed in the non‐GP ablation group or in the overall cohort (Supporting Information S1: Figure S2), a pattern compatible with the hypothesis that clinical indicators of more advanced atrial substrate may be more closely related to early recurrence after autonomic modulation.

**Figure 2:**
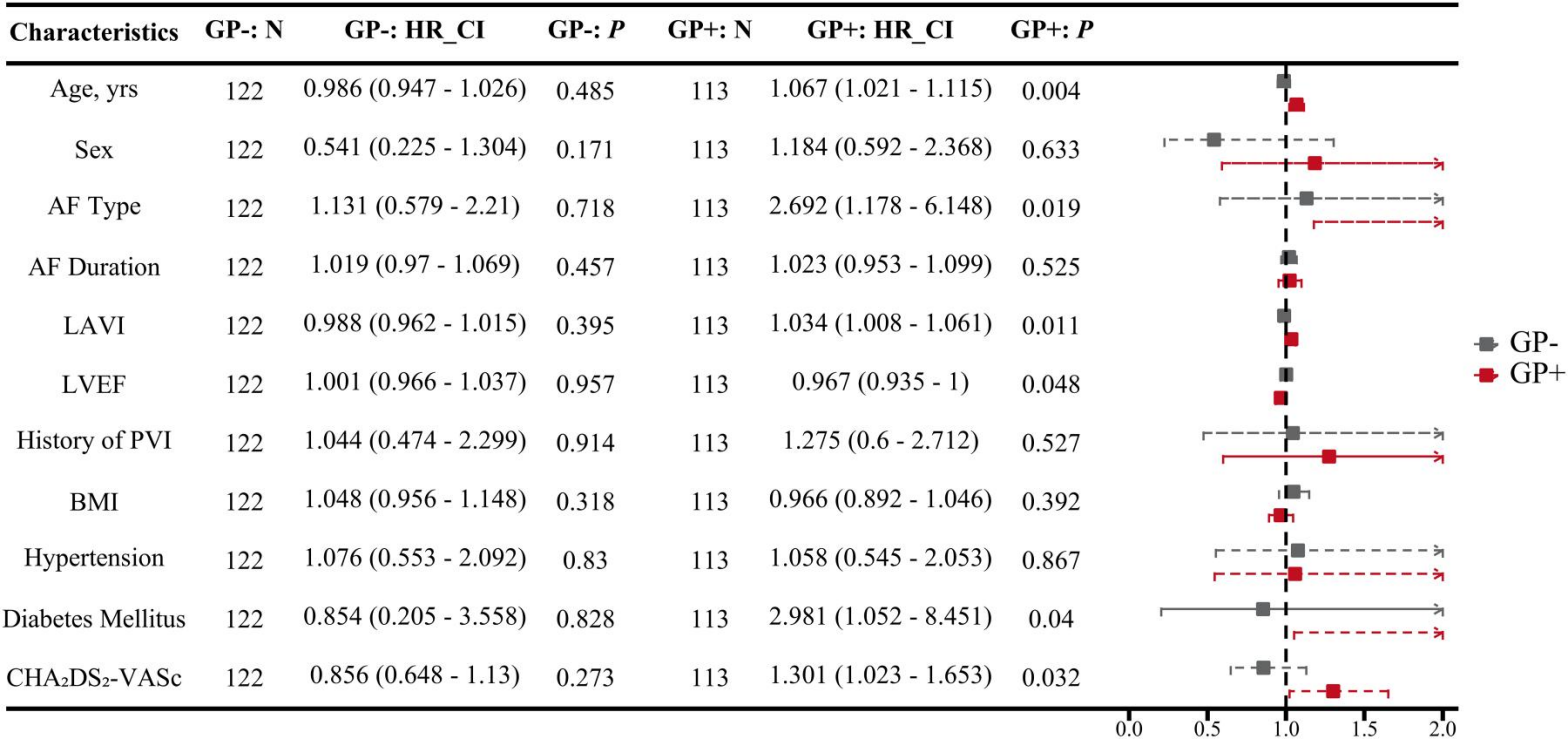
Univariate Cox regression analysis for predictors of early recurrence. Subgroup analysis by GP ablation status. HR and 95% CI are depicted. The dashed vertical line marks HR = 1. GP = ganglionated plexus; HR = hazard ratio; CI = confidence interval; AF = atrial fibrillation; LAVI = left atrial volume index; LVEF = left ventricular ejection fraction; PVI = pulmonary vein isolation; BMI = body mass index.

In multivariable Cox models, interaction terms suggested effect modification between GP ablation and age (per 1‐year, HR 1.08; 95% CI 1.01–1.14; *p* = 0.016) as well as LAVI (per 1 mL/m^2^, HR 1.04; 95% CI 1.01–1.09; *p* = 0.026), indicating that the associations of these variables with early recurrence may differ according to GP ablation status.

### 3.4 Early Recurrence After GP Ablation and Risk of Late Recurrence

The association between early and late AF recurrence differed between the GP and non‐GP groups (Figure 3A and Supporting Information S1: Table S1). Within each group, we assessed whether early recurrence was associated with subsequent late recurrence. In the GP ablation group, early recurrence within 0–90 days was associated with an increased risk of late recurrence (HR = 2.60, 95% CI 1.33–5.10, *p* = 0.005). No corresponding association was observed in the control group (HR = 0.98, 95% CI 0.49–1.95, *p* = 0.946; Figure 3A). A similar pattern was observed within the first 30 days: in the GP group, early recurrence was associated with late recurrence (HR = 2.63, 95% CI 1.28–5.39, *p* = 0.009), whereas in the non‐GP group, this association was not observed (HR = 0.51, 95% CI 0.20–1.30, *p* = 0.156). No clear association was observed for recurrence during days 30–60. Recurrence during days 60–90 was associated with late recurrence in both treatment groups, although these estimates were based on very small numbers and should be interpreted cautiously. There was no apparent evidence that this association differed according to GP‐ablation status.

**Figure 3:**
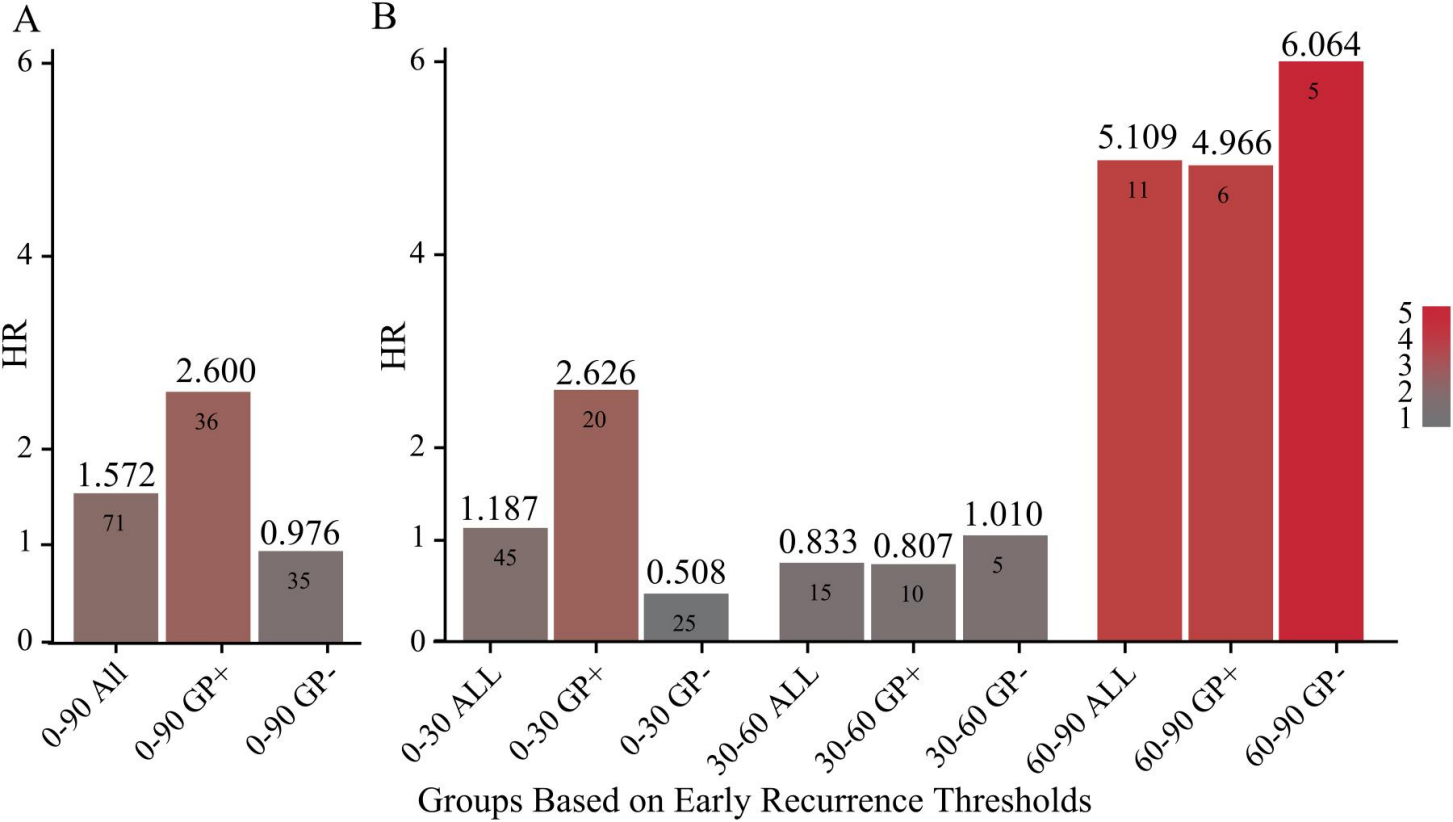
Hazard ratios (HRs) for late recurrence based on different early recurrence time intervals: (A) 0–90 days and (B) 0–30, 30–60, and 60–90 days. HRs are shown for the overall cohort (All), patients with GP ablation (GP + ), and those without GP ablation (GP − ). Numbers above each bar represent the HR, and numbers inside each bar denote the number of patients with early recurrence in that interval.

Between‐group comparisons assessed whether the strength of the association between early and late recurrence differed for GP vs. non‐GP patients. Among patients with recurrence within 30 days, the GP ablation group had a higher estimated risk of late recurrence compared with controls (HR = 3.41, 95% CI 1.18–9.82, *p* = 0.023; Figure 4A). For recurrence within 0‐90 days, this between‐group difference was not statistically significant (HR = 1.69, 95% CI 0.79–3.60, *p* = 0.177; Figure 4B), although late recurrence was numerically more frequent in the GP ablation group. In line with these findings, Cox models including a treatment × early recurrence interaction term were compatible with effect modification during the 0‐90‐day period (interaction HR 2.75; 95% CI 1.05–7.24; *p* = 0.040), with a stronger estimate for 0–30 days (interaction HR 5.34; 95% CI 1.64–17.40; *p* = 0.005). Because these findings derive from subgroup and interaction analyses without multiple‐comparison adjustment, they should be interpreted cautiously.

**Figure 4:**
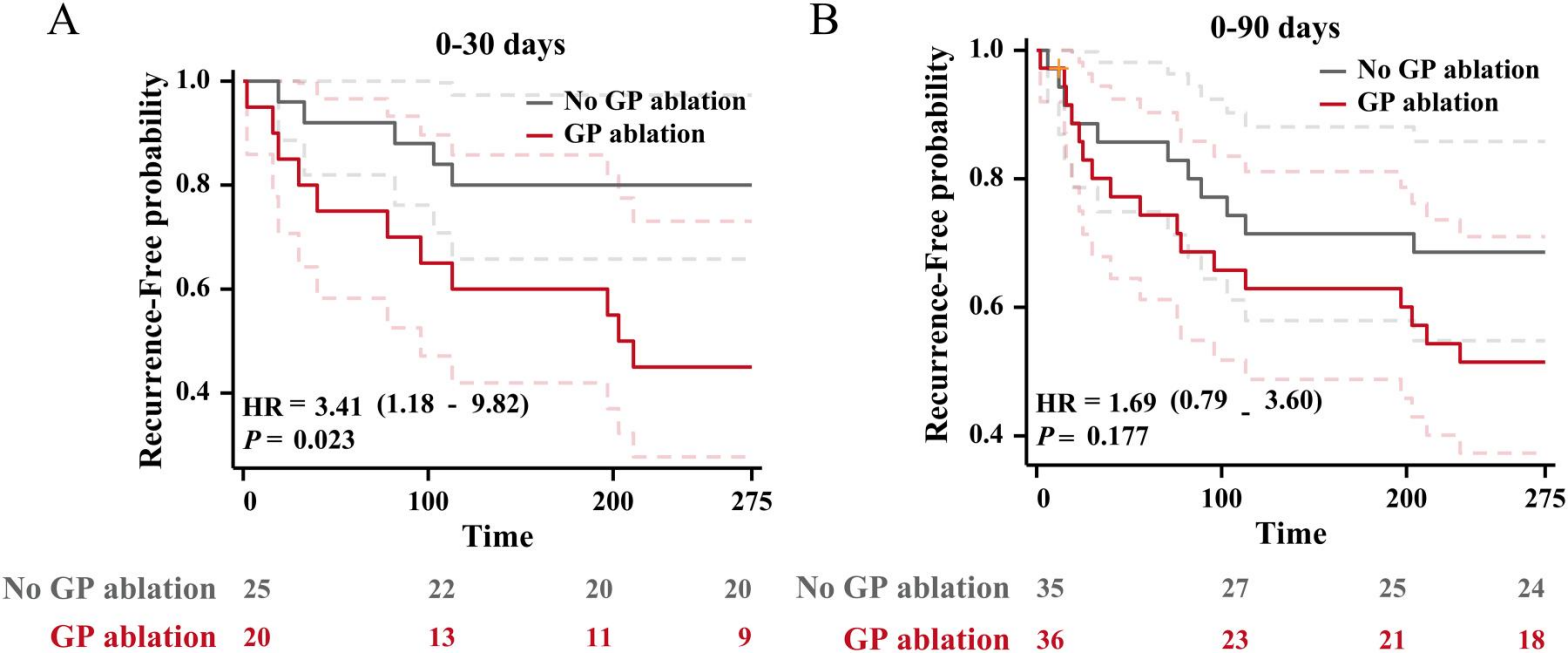
Kaplan–Meier curves comparing late recurrence between GP+ and GP− patients with early recurrence. (A) Early recurrence within 0–30 days; (B) Early recurrence within 0–90 days. An association with higher late recurrence was observed in the GP+ group for the 0–30 day interval. HR, hazard ratio.

### 3.5 Transcriptomic Correlates of Early and Late Recurrence Suggest Autonomic and Inflammatory/Fibrotic Pathways

Compared with patients without early recurrence, those with early recurrence had pathways related to autonomic nervous system regulation among the top upregulated pathways, including the neuronal system, synaptic transmission, and neurotransmitter release pathways (Supporting Information S1: Figure S3A–C). In contrast, patients with late recurrence, compared with those without late recurrence, had top upregulated pathways associated with inflammation and fibrosis, such as interleukin signaling (IL‐10, IL‐4, and IL‐13) and extracellular matrix organization (Supporting Information S1: Figure S3D–F). These transcriptomic findings should be viewed as an exploratory biological context rather than direct evidence of postoperative autonomic or structural mechanisms.

## 4 Discussion

### 4.1 Main Findings

GP ablation was associated with a delayed onset of the first early recurrence after thoracoscopic ablation of advanced AF, but not with a lower rate of early AF recurrence. Additionally, in patients with GP ablation, early recurrence was associated with late recurrence, whereas this association was not observed in patients without GP ablation. These findings raise the possibility that GP ablation may modify the clinical meaning of early recurrence. They should not be interpreted as evidence that GP ablation improves clinical outcomes, but rather as providing insight into how autonomic modulation may affect trigger‐driven early recurrence.

Our findings support an effect of GP ablation on the temporal pattern of early recurrence during the blanking period and are compatible with a role for autonomic mechanisms in early recurrence. Although we cannot provide longitudinal direct evidence of GP function during follow‐up, autonomic effectors were carefully assessed during the GP ablation procedure: high‐frequency stimulation was used to elicit vagal responses (i.e., atrioventricular block, sinus bradycardia, or sinus block), and elimination of these responses upon repeat high‐frequency stimulation was confirmed in patients randomized to GP ablation. Moreover, heart‐rate changes on Holter monitoring during follow‐up in the original AFACT study further supported denervation in the randomized GP ablation group [9]. Nevertheless, the present post hoc analysis cannot establish whether autonomic modulation caused the observed recurrence pattern, nor can it define the completeness or durability of denervation. The transcriptomic findings provide biological context, with early recurrence showing enrichment of pathways related to neuronal signaling and late recurrence showing enrichment of inflammatory and fibrotic pathways. Because left atrial appendage tissue was obtained intraoperatively, these data should primarily be interpreted as reflecting pre‐existing molecular substrate rather than postoperative molecular changes. As illustrated by the time‐varying HR in Supporting Information S1: Figure S1, the estimated effect of GP ablation appeared most pronounced during the first 2 weeks. This temporal pattern may explain why GP ablation did not reduce the overall 0‐90‐day early recurrence rate: a possible early reduction or delay in recurrence could be offset by later recurrences within the blanking period. The predominance of early recurrences within the first 30 days is consistent with the concept that transient postoperative factors, including inflammation, acute lesion effects, and autonomic imbalance, may be most relevant during the earliest phase of the blanking period. However, the treatment‐by‐time interaction was borderline (*p* = 0.054), and this finding should therefore be interpreted as exploratory. Another possible explanation is that GP ablation may induce additional scar formation, which is pro‐inflammatory and potentially arrhythmogenic, thereby counteracting the potential beneficial effects of GP ablation [15, 16]. Additionally, no clear association between early and late recurrence was observed for recurrences occurring during days 30–60, whereas recurrences during days 60–90 were associated with late recurrence in both the GP‐ablation and control groups. Although based on very small numbers and therefore requiring cautious interpretation, this temporal pattern is consistent with our previous study, in which later‐occurring early recurrence showed a stronger association with subsequent late recurrence [5].

We postulate the following hypothesis for interpretation of these results (Figure 5): vulnerability for early AF recurrence may reflect the interplay between transient postoperative factors and a more permanent pre‐existing arrhythmogenic substrate. Part of the transient component may relate to autonomic innervation, which GP ablation is expected to attenuate. Under this model, patients with limited underlying substrate may no longer reach the threshold for AF recurrence early after ablation, whereas patients with a more advanced substrate may still experience early recurrence, albeit at a later time. Early recurrence occurring despite autonomic modulation may therefore signal a greater contribution of the underlying arrhythmogenic substrate, which could explain its stronger association with late recurrence. In patients without GP ablation, early recurrence may arise from either transient postoperative factors or pre‐existing substrate, whichever reaches the threshold for AF first. This could explain the earlier onset during the blanking period and the weaker association with late recurrence.

**Figure 5:**
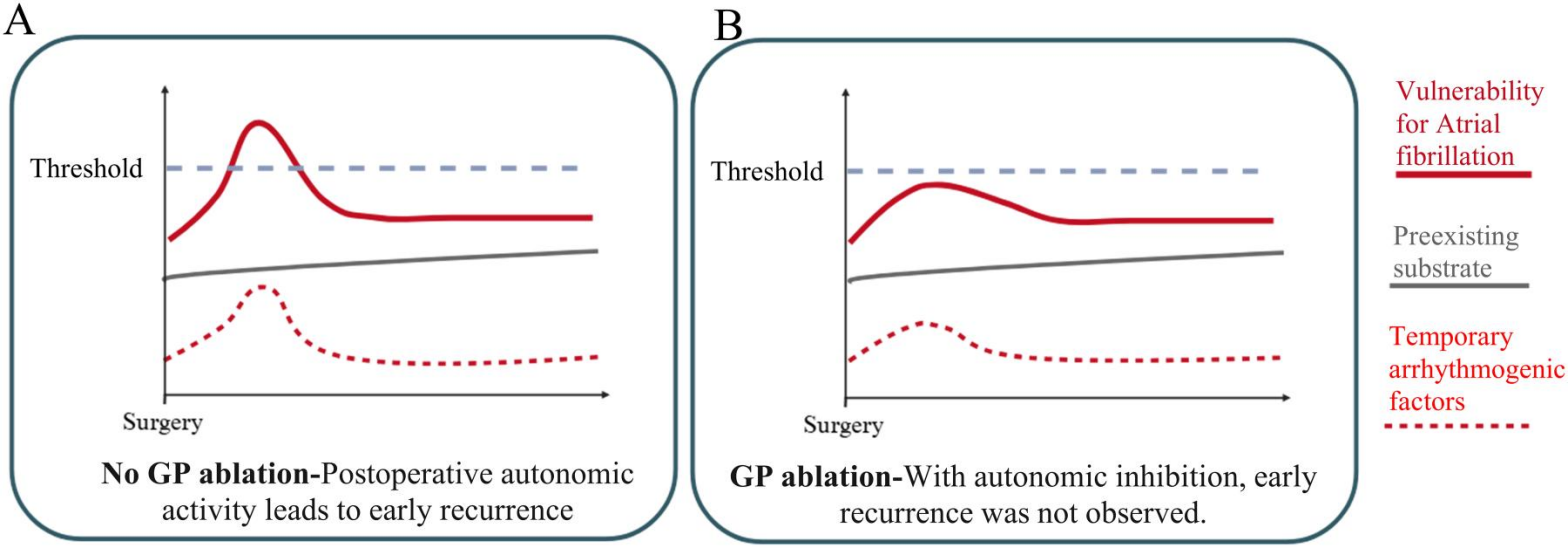
Proposed hypothesis of autonomic modulation and early recurrence. (A) Without suppression of autonomic innervation, pre‐existing substrate and transient postoperative triggers may combine to exceed the threshold for AF recurrence. (B) With suppression of autonomic innervation, the autonomic trigger component may be attenuated, so residual early recurrence may be more dependent on pre‐existing substrate. This model is speculative and requires prospective validation.

These findings may also be relevant when interpreting AF recurrence in patients receiving sympatholytic or vagolytic drugs after ablation [17, 18]. For example, early recurrence in patients treated with postoperative β‐blockers might reflect a more substrate‐driven mechanism than early recurrence in patients without such therapy. This extrapolation remains speculative and should not be used to guide therapy without prospective validation. More broadly, the mechanisms underlying AF recurrence may vary across postprocedural stages, with autonomic, inflammatory, fibrotic, and other factors predominating at different times. A multifactorial approach may therefore be useful for future studies of patients with early AF recurrence [19].

### 4.2 Study Limitations

Several limitations should be acknowledged. First, rhythm monitoring during the blanking period was based on scheduled follow‐up and symptom‐driven recordings rather than continuous monitoring. This may have led to under‐detection of asymptomatic early recurrence and imprecision in the timing of the first documented recurrence. Because this was a randomized comparison and the monitoring strategy was applied to both treatment arms, systematic between‐group detection bias is less likely; however, differences in asymptomatic recurrence burden cannot be excluded. Second, the sample size was limited, particularly in the 30–60 day and 60–90 day intervals, which reduced the precision and robustness of the estimates. Third, all patients received colchicine for 30 days postoperatively, and antiarrhythmic drugs were continued for 3 months. These therapies were protocolized and applied in both randomized groups, and are therefore unlikely to explain between‐group differences, but they may have influenced recurrence rate, recurrence timing, and the observed associations. We cannot exclude the possibility that colchicine or antiarrhythmic medication interacted differently with autonomically modulated atria. Fourth, autonomic modulation was not continuously or comprehensively assessed during follow‐up. Although the AFACT intervention included procedural evidence of autonomic modulation through high‐frequency stimulation‐induced vagal responses and confirmation of their elimination after GP ablation, and heart‐rate changes reported in the original trial provide indirect support for denervation, these data do not establish the completeness or durability of denervation throughout the blanking period, nor do they prove that the observed recurrence pattern was mechanistically caused by autonomic modulation. Fifth, multiple comparisons were not adjusted for. The possibility of type I error inflation, especially in subgroup and interaction analyses, cannot be excluded; therefore, the findings should be interpreted as exploratory and warrant confirmation in independent datasets. Finally, extrapolation of these findings to other ablation settings should be made with caution. The data were obtained in patients with advanced AF, namely persistent AF, enlarged left atria, or prior failed catheter ablation, undergoing thoracoscopic epicardial ablation with extensive lesion sets. This setting differs from catheter‐based GP ablation, catheter‐based autonomic modulation, and cardioneural ablation, and the present findings should not be directly generalized to those approaches.

## 5 Conclusion

Although adjunctive thoracoscopic GP ablation cannot be recommended for routine treatment of AF, in this randomized post hoc analysis it was associated with later onset of first early recurrence after thoracoscopic ablation of advanced AF. Early recurrence in GP‐ablated patients was associated with a higher risk of late AF recurrence. These exploratory findings support a hypothesis‐generating model in which early recurrence reflects both transient autonomic triggers and pre‐existing substrate, and in which residual early recurrence after GP ablation may be more substrate‐related. The findings should be used to inform future studies rather than to justify routine GP ablation in clinical practice.

## Author Contributions

Z.D. and J.R.d.G. conceived the study, performed the analyses, and drafted the manuscript. Z.D. was responsible for data collection and initial processing. M.T. contributed to the study design, assisted in data collection, and was actively involved in revising the initial draft of the manuscript. All other co‐authors contributed to the critical review, provided intellectual input to improve the manuscript, and approved the final version. J.R.d.G. supervised the project, reviewed the drafts of the manuscript, and provided funding support.

## Ethics Statement

This study was approved by the Institutional Review Board of Amsterdam University Medical Center Ethical Review Committee (ClinicalTrials.gov Identifier: NCT01091389).

## Consent

Informed consent was obtained from all participants included in the study.

## Conflicts of Interest

J.R. de Groot is supported by Information Technology for European Advancement (ITEA4, grant number 21026), CVON/Dutch Heart Foundation grant 01‐002‐2022‐0118 (EmbRACE), and the ERA4Health PERSUADE consortium grant. J.R. de Groot has received research grants (through the institution) from Atricure Inc., Bayer, Boston Scientific, Daiichi Sankyo, Johnson & Johnson, Medtronic, and Philips. He has received honoraria, speaker, or consultancy fees from AtriaN Medical, Atricure Inc., Bayer, Berlin Chemie, Daiichi Sankyo, Johnson & Johnson, Menarini, Medtronic, Novartis, and Servier.

## Supporting information


Supporting File

## Data Availability

The data underlying this article will be shared upon reasonable request.
