# Supplementary material for: Impact of Ganglionated Plexus Ablation on Early Recurrence After Thoracoscopic Atrial Fibrillation Ablation: A Post Hoc Analysis of the AFACT Study
Source: J Cardiovasc Electrophysiol. 2026 Oct 6;37(11):2722–9. doi: 10.1111/jce.70517 (PMC13768774; doi:10.1111/jce.70517)
Supplement: Supplementary file 1 [file JCE-37-2722-s001.docx]

**SUPPLEMENTARY MATERIALS**

**Impact of Ganglionated Plexus Ablation on Early Recurrence After Thoracoscopic Atrial Fibrillation Ablation: A Post Hoc Analysis of the AFACT Study**

Zhenyu Dong, MD^1,2†^, Marc M. Terpstra, MD ^1,2†^, Jolien Neefs, MD, PhD^1,2^, Nicoline W.E. van den Berg, MD, PhD^1,2^, Femke R. Piersma, MSc^1,2^, Sebastien P.J. Krul, MD, PhD^3^, Wouter R. Berger, MD, PhD^4^, WimJan P. van Boven, MD, PhD^1,2^, Jonas S.S.G. de Jong, MD, PhD^4^, Ruben Coronel, MD, PhD^1,2^, Joris R. de Groot, MD, PhD^1,2*^, Antoine H.G. Driessen, MD, PhD^1,2^

1 Amsterdam UMC, location University of Amsterdam, Department of Cardiology, Amsterdam, the Netherlands.

2 Amsterdam Cardiovascular Sciences, Heart Failure and Arrhythmias, Amsterdam, the Netherlands.

3 Department of Cardiology, Heart Center, Isala, Zwolle, The Netherlands.

4 Department of Cardiology, Onze Lieve Vrouwe Gasthuis (OLVG), Amsterdam, The Netherlands.

**†** These authors contributed equally to this work.

## Methods

**RNA sequencing data analyses**

We included patients who underwent thoracoscopic AF ablation at Amsterdam UMC, Meibergdreef, the Netherlands. In addition to those enrolled in the AFACT study, patients from the MARK-AF registry (NL50069.018.14) were also included.^2^ All patients from the MARK-AF registry underwent the same standalone thoracoscopic ablation procedure as those included in the AFACT study. During surgery, left atrial appendage tissue was obtained from 42 patients, including 2 patients from the control group of the AFACT study and the remaining patients from the MARK-AF registry, and subjected to bulk RNA sequencing.^1^ Eleven patients experienced late recurrence, and eight developed early recurrence; these categories were not mutually exclusive, and some patients experienced both. The detailed RNA sequencing procedures have been described previously.^1^ In short, reads were aligned to the human genome (GRCh38, v94) using HISAT2, and uniquely mapped reads were counted with HTSeq. Lowly expressed genes were filtered out, and data were normalized by TMM (edgeR) and log2-transformed with voom. Differential expression was analyzed with limma–voom including age and sex as covariates, using Benjamini–Hochberg FDR correction. Comparisons were made between patients with and without early or late recurrence.

**Gene Set Enrichment Analyses**

Gene Set Enrichment Analyses (GSEA) followed the procedure of Subramanian et al.^3,4^ First, genes were ranked by multiplying the sign of the log₂ fold‑change with the negative log₁₀ of the Benjamini–Hochberg–adjusted P value.^5^ Canonical pathway gene sets were taken from MSigDB version 2022.1 (C2: CP collection) and restricted to sets containing 10–500 genes. Enrichment scores and normalized enrichment scores (NES) were computed using the pre-ranked GSEA implemented in clusterProfiler (function GSEA), with 1,000 gene-set permutations to estimate the null distribution. Pathways were considered significantly enriched when the FDR q‑value was below 0.25. Significant pathways were visualized with enrichment plots and bar plots to illustrate the magnitude and direction of enrichment.

## Table S1

Association between early recurrence (ER) and late AF recurrence (LR), shown as Cox HRs (95% CIs) across ER time windows (0–30, 30–60, 60–90, 0–90 days) and GP ablation strata (GP+, GP−), with counts provided.

| Time Window | Group | HR | 95% CI | *P* | N | ER N | noER N | ER_LR_N | noER_LR_N | |
| --- | --- | --- | --- | --- | --- | --- | --- | --- | --- | --- |
| 0-90 days | All | 1.572 | 0.983-2.516 | 0.059 | 235 | 71 | 164 | 28 | 46 |  |
| 0-90 days | GP+ | 2.600 | 1.326-5.098 | 0.005 | 113 | 36 | 77 | 17 | 17 |  |
| 0-90 days | GP- | 0.976 | 0.488-1.955 | 0.946 | 122 | 35 | 87 | 11 | 29 |  |
| 0-30 days | All | 1.187 | 0.683-2.065 | 0.544 | 235 | 45 | 190 | 16 | 58 |  |
| 0-30 days | GP+ | 2.626 | 1.279-5.394 | 0.009 | 113 | 20 | 93 | 11 | 23 |  |
| 0-30 days | GP- | 0.508 | 0.199-1.296 | 0.156 | 122 | 25 | 97 | 5 | 35 |  |
| 30-60 days | All | 0.833 | 0.302-2.301 | 0.725 | 189 | 15 | 174 | 4 | 54 |  |
| 30-60 days | GP+ | 0.807 | 0.189-3.442 | 0.772 | 92 | 10 | 82 | 2 | 21 |  |
| 30-60 days | GP- | 1.010 | 0.242-4.211 | 0.989 | 97 | 5 | 92 | 2 | 33 |  |
| 60-90 days | All | 5.109 | 2.395-10.896 | <0.001 | 174 | 11 | 163 | 8 | 46 |  |
| 60-90 days | GP+ | 4.966 | 1.652-14.930 | 0.004 | 82 | 6 | 76 | 4 | 17 |  |
| 60-90 days | GP- | 6.064 | 2.106-17.467 | 0.001 | 92 | 5 | 87 | 4 | 29 |  |

Abbreviations: HR, hazard ratio; P, p-value; N, number; ER, early recurrence; noER, no early recurrence; LR, late recurrence; GP, ganglionated plexus ablation.

## Figure S1

Figure S1. Time-varying hazard ratio HR(t) for AF recurrence (GP ablation vs non-GP). Red line: HR(t) from a single time-dependent Cox model (treatment × log(time)); shaded band: 95% CI; dashed line: HR = 1.

## Figure S2

Figure S2. Univariate Cox regression analysis for predictors of early recurrence. Hazard ratios (HR) and 95% confidence intervals (CI) are depicted. The dashed vertical line marks HR=1.

## Figure S3

Figure S3. Gene set enrichment analysis (GSEA) of early and late recurrence.

(A) Top enriched pathways in early recurrence, including neuronal signaling–related pathways. (B–C) Enrichment plots of representative nervous system-related pathways: Neuronal System (B) and Neurotransmitter Release Cycle (C). (D) Top enriched pathways in late recurrence, showing upregulation of inflammation and fibrosis-related processes. (E–F) Enrichment plots for Interleukin-10 signaling (E) and Interleukin-4 and -13 signaling (F). Normalized enrichment score (NES), adjusted p-value, and FDR q-value are indicated.

## References

1.	Driessen AHG, Berger WR, Krul SPJ, et al.: Ganglion Plexus Ablation in Advanced Atrial Fibrillation: The AFACT Study. J Am Coll Cardiol 2016; 68:1155–1165.

2.	van den Berg NWE, Kawasaki M, Fabrizi B, et al.: Epicardial and endothelial cell activation concurs with extracellular matrix remodeling in atrial fibrillation. Clin Transl Med 2021; 11:e558.

3.	Reimand J, Isserlin R, Voisin V, et al.: Pathway enrichment analysis and visualization of omics data using g:Profiler, GSEA, Cytoscape and EnrichmentMap. Nat Protoc 2019; 14:482–517.

4.	Subramanian A, Tamayo P, Mootha VK, et al.: Gene set enrichment analysis: A knowledge-based approach for interpreting genome-wide expression profiles. Proceedings of the National Academy of Sciences Proceedings of the National Academy of Sciences, 2005; 102:15545–15550.

5.	van den Berg NWE, Neefs J, Kawasaki M, et al.: Extracellular matrix remodeling precedes atrial fibrillation: Results of the PREDICT-AF trial. Heart Rhythm 2021; 18:2115–2125.
